# Serum Visfatin Levels in Nonalcoholic Fatty Liver Disease and Liver Fibrosis: Systematic Review and Meta-Analysis

**DOI:** 10.3390/jcm10143029

**Published:** 2021-07-07

**Authors:** Abdulrahman Ismaiel, Daniel-Corneliu Leucuta, Stefan-Lucian Popa, Dan L. Dumitrascu

**Affiliations:** 1 2nd Department of Internal Medicine, “Iuliu Hatieganu” University of Medicine and Pharmacy, 400006 Cluj-Napoca, Romania; abdulrahman.ismaiel@yahoo.com (A.I.); popa.stefan@umfcluj.ro (S.-L.P.); ddumitrascu@umfcluj.ro (D.L.D.); 2 Department of Medical Informatics and Biostatistics, “Iuliu Hatieganu” University of Medicine and Pharmacy, 400349 Cluj-Napoca, Romania

**Keywords:** visfatin, adipokines, NAFLD, hepatic steatosis, liver fibrosis, NASH

## Abstract

(1) Background: Recently, adipokines, including visfatin, have been studied in nonalcoholic fatty liver disease (NAFLD). Several studies evaluated visfatin levels in NAFLD, the presence and severity of hepatic steatosis, liver fibrosis, lobar inflammation, nonalcoholic steatohepatitis (NASH), and gender differences. However, inconclusive results have been reported. Accordingly, we performed a systematic review and meta-analysis, aiming to address these gaps in evidence. (2) Methods: We performed a systematic electronic search on PubMed, EMBASE, and Cochrane Library using predefined keywords. Diagnosis of NAFLD by liver biopsy or imagistic investigations was accepted. Full articles satisfying our inclusion and exclusion criteria were included. NHLBI quality assessment tools were used to evaluate included studies. The principal summary outcome was the mean difference in visfatin levels. (3) Results: There were 21 studies involving 1923 individuals included in our qualitative assessment, while 14 studies were included in the quantitative assessment. No statistical significance was found assessing visfatin levels in NAFLD [3.361 (95% CI −0.175–6.897)], simple steatosis [7.523 (95% CI −16.221–31.267)], hepatic steatosis severity [−0.279 (95% CI −1.843–1.285)], liver fibrosis [4.133 (95% CI −3.176–11.443)], lobar inflammation [0.358 (95% CI −1.470–2.185)], NASH [−2.038 (95% CI −6.839–2.763)], and gender [(95% CI −0.554–0.556)]. (4) Conclusions: In conclusion, visfatin levels are not associated with NAFLD, presence or severity of hepatic steatosis, liver fibrosis, lobar inflammation, NASH, and gender. However, due to the limited methodological quality of the included studies, results should be interpreted with caution.

## 1. Introduction

Nonalcoholic fatty liver disease (NAFLD) is primarily a liver pathology associated with structural and functional liver modifications, increased liver-related morbidity and mortality due to possible progression to cirrhosis, liver failure, and ultimately, hepatocellular carcinoma, as well as several extrahepatic manifestations [1,2,3,4]. Until the present, NAFLD remains without currently approved therapies [5,6,7]. The worldwide prevalence of associated metabolic diseases such as NAFLD, type 2 diabetes mellitus, dyslipidemia, and obesity has dramatically increased over the last decades [8].

The development and progression of NAFLD are based on a complex and multifactorial mechanism, explained by a recent hypothesis known as the “multiple-hit model” that has now been more widely accepted, describing a prominent metabolic dysfunction due to several genetic and environmental interactions, in addition to changes in crosstalk between several organs and tissues such as adipose tissue, liver, pancreas, and gut [9].

Adipose tissue is considered because of highly active endocrine tissue-producing peptides known as adipokines that exert autocrine, paracrine, and endocrine functions. Despite conflicting evidence, adipokines have gained increasing interest in several obesity-related diseases, including NAFLD [8,10]. However, the pathogenic effects exerted by adipokines in NAFLD remain under investigation. 

Among these adipokines is visfatin, a highly conserved 52-kDa protein that is found in all living species, also known as nicotinamide phosphoribosyltransferase (NAMPT) and pre-B-cell colony-enhancing factor 1 (PBEF-1). Visfatin has several main sources, including adipocytes, lymphocytes, monocytes, neutrophils, hepatocytes, and pneumocytes [11]. Various pathways affected by visfatin include oxidative stress response, apoptosis, lipid, and glucose metabolism, as well as insulin resistance and inflammation, possibly playing a role in the pathogenesis of NAFLD [12,13,14]. The expression of visfatin is regulated by several cytokines such as tumoral necrosis factor-alpha (TNF α), interleukin-6 (IL-6), and lipopolysaccharide that are known to promote insulin resistance [15]. Furthermore, increased visfatin levels were found to be associated with atherosclerotic disease and coronary artery disease, pathologies demonstrated to be among the main mortality causes in NAFLD [16,17,18,19].

Although several studies have evaluated the role of visfatin and its exerted effects on hepatic steatosis, fibrosis, and inflammation in NAFLD, current evidence remains inconclusive with conflicting results, limiting our understanding of the physiological and pathophysiological roles of visfatin in NAFLD. Therefore, we conducted the first systematic review and meta-analysis to the best of our knowledge, evaluating the association between visfatin and NAFLD, the presence and severity of hepatic steatosis, liver fibrosis, lobar inflammation, and NASH, in addition to possible gender differences.

## 2. Materials and Methods

We wrote this systematic review and meta-analysis according to the Preferred Reporting Items for Systematic Reviews and Meta-Analyses (PRISMA) checklist 2009 [20].

### 2.1. Data Sources and Search Strategy

We aimed to review all the current evidence published on PubMed, EMBASE, and Cochrane Library reporting observational studies assessing the role of visfatin in NAFLD, the presence and severity of hepatic steatosis, liver fibrosis, lobar inflammation, and NASH, as well as gender differences. A detailed description of the performed search strategy is provided in Supplementary Material S1. In order to minimize results bias, a manual search was conducted for relevant missed publications by searching the references of included articles. We searched for published articles from inception up to 21 April 2020 without applying any search filters or restrictions to duration, country, or language. Subsequently, we performed a screening evaluation by assessing titles and abstracts for appropriateness. Selected articles fulfilling the inclusion and exclusion criteria underwent a full-text review. Eligibility of the evaluated studies and data extraction from eligible studies was performed by two authors (A.I. and D.-C.L) independently while resolving any discrepancies by mutual consensus.

#### Eligibility Criteria

Inclusion criteria of original articles were as follows: (1) full article studies of observational cohort population-based/ hospital-based, cross-sectional, or case-control designs that assessed visfatin effects on hepatic steatosis, inflammation, and fibrosis in NAFLD; (2) hepatic steatosis evaluated using liver biopsy or imaging techniques such as ultrasonography, computed tomography (CT), and magnetic resonance imaging (MRI) in the absence of other secondary causes of hepatic steatosis or significant alcohol consumption; (3) liver fibrosis evaluated using liver biopsy or transient elastography (FibroScan); (4) human studies only; and (5) studies published in English, German, or Romanian languages. 

Exclusion criteria were as follows: (1) significant alcohol consumption or presence of other secondary causes of hepatic steatosis; (2) confirmed hepatitis virus of any etiology; (3) other known causes of CLD; (4) confirmed cirrhosis of any etiology; (5) subjects with end-stage liver disease awaiting liver transplantation or who received a liver transplant; and (6) editorials, letters to the editor, case reports, conference abstracts, literature and systematic reviews, practice guidelines, commentaries, and abstracts published without an entire article.

### 2.2. Risk of Bias Assessment in Individual Studies

The risk of bias in individual studies was evaluated using quality assessment tools from the National Heart, Lung, and Blood Institute (NHLBI) [21]. Two tools were used for observational cohort and cross-sectional studies as well as case-control studies. These evaluation tools were used in order to assess bias risk and internal validity in individual studies in a similar manner. Two authors (A.I. and D.-C.L) performed the evaluation independently. In case of disagreement, a consensus was reached through a discussion.

### 2.3. Summary Measures and Synthesis of Results

The principal summary outcome was the mean difference (MD) of visfatin levels. The data analyses of the systematic review and meta-analysis were conducted using R with Metafor package (OpenMeta [Analyst]) [22,23]. The χ2 based Q-test and I^2^ were used to evaluate between-study heterogeneity. The random-effects model and MD were used for the analysis of estimated total effect size. We calculated the mean and standard deviation (SD) in studies that reported medians and interquartile ranges, as well as ranges, in addition to combining groups in studies that had several subgroups of NAFLD patients or control subjects without a total group, according to the Cochrane Handbook recommendations. Subgroup analysis was performed according to the presence of simple steatosis, moderate to severe steatosis, NASH, lobar inflammation, liver fibrosis, and gender differences according to the available values from the extracted data present in the included studies. We reported the data from each study as the estimated MD with 95% CI. A *p*-value < 0.05 was considered statistically significant.

### 2.4. Risk of Bias Across Studies

We did not perform an overall assessment of the risk of bias across studies in our systematic review and meta-analysis as it is not recommended according to the Cochrane Handbook, mainly due to unavailable data from included studies regarding several outcomes that might be critical, as well as variations of critical outcomes from setting to setting due to factors such as societal values or baseline risk.

## 3. Results

### 3.1. General Results

**Figure 1:**
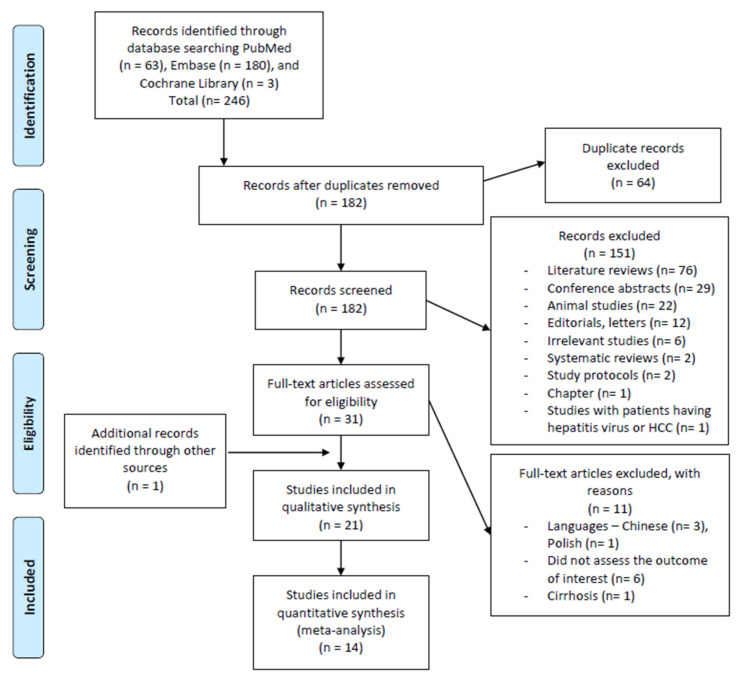
The PRISMA flow diagram for the search and selection processes of this systematic review and meta-analysis.

The initial search yielded 246 articles (PubMed = 63 articles, EMBASE = 180 articles, Cochrane Library = 3 articles) as demonstrated in Figure 1. A total of 64 studies were detected as duplicates and removed. After the removal of duplicates, 182 articles were evaluated for inclusion and exclusion criteria fulfillment through assessing the titles and abstracts. Screening demonstrated the following were the results: (1) 78 reviews (literature reviews *n* = 76, systematic reviews *n* = 2), (2) 29 conference abstracts, (3) 22 studies conducted on animals, (4) 12 letters/editorials, (5) 2 study protocols, (6) 1 chapter, (7) 1 study including patients with viral hepatitis or hepatocellular carcinoma, (8) 6 other irrelevant studies to this review topic, and (9) 20 article abstracts that met the primary criteria. A total of 151 studies were excluded during the first screening. We performed a thorough reading and evaluation of the full-texts for further eligibility assessment for the remaining 31 articles. Of these articles, 11 were excluded with reasons as follows: (1) four articles conducted in languages other than English, German, or Romanian (Chinese Language = three articles, Polish language = one article) [24,25,26,27], (2) six articles did not assess the outcome of interest [28,29,30,31,32,33], (3) one article involved patients with toxic cirrhosis and primary biliary cirrhosis [34]. Upon reviewing the references of included studies manually, one relevant article was recognized and included in our analysis. The total number of articles included in the qualitative synthesis was 21 articles, out of which 14 articles were included in the quantitative synthesis [35,36,37,38,39,40,41,42,43,44,45,46,47,48,49,50,51,52,53,54,55].

### 3.2. Study Characteristics

A summary of the main characteristics of included studies is shown in Supplementary Table S1. This systematic review and meta-analysis included a total number of 1923 individuals (1022 individuals in case-control studies; 495 individuals in cross-sectional studies, out of which 54 subjects are overlapping with another study of cross-sectional design; and 460 individuals in prospective cohort studies). The sex distribution was higher for males (females–882 (46%), males–1041 (54%)). NAFLD was present in 1135 subjects (54%) out of the total study sample. 

Ten studies had a cross-sectional study design, whereas nine had a case-control design and two had a prospective cohort design. Eight studies were undertaken in Europe (Spain *n* = 2, Turkey n = 2, Germany *n* = 1, Norway *n* = 1, Poland *n* = 1, Greece *n* = 1), eight in the Middle East (Iran *n* = 7, Egypt *n* = 1), three studies in Asia (China *n* = 1, Korea *n* = 1, India *n* = 1), and two studies in the USA (*n* = 2).

### 3.3. Definition of NAFLD

**Figure 2:**
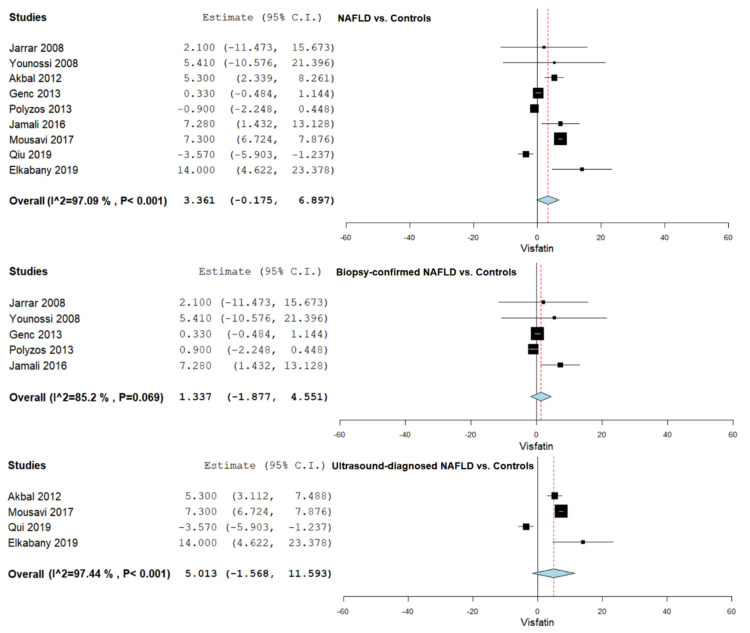
Studies evaluating serum visfatin levels in NAFLD vs. controls, biopsy-proven NAFLD vs. controls, and ultrasound evaluated hepatic steatosis vs. controls.

Hepatic steatosis was assessed using liver biopsy for diagnosing NAFLD in most studies (*n* = 11) [35,36,37,38,39,40,42,43,44,45,46,47,48], while the remaining studies used ultrasonography [41,49,50,51,52,53,54,55]. Moreover, four studies that used ultrasonography to evaluate hepatic steatosis used Fibroscan in order to assess liver stiffness [49,50,51,53]. Figure 2 summarizes the obtained meta-analysis results comparing serum visfatin levels in NAFLD vs. controls, biopsy-proven NAFLD vs. controls, and ultrasound evaluated hepatic steatosis vs. controls.

### 3.4. Serum Visfatin Levels in NAFLD vs. Controls

Serum visfatin levels were evaluated in a total of nine studies comparing NAFLD patients with control subjects [35,36,41,44,45,46,52,53,55]. The pooled studies for the analysis assessing serum visfatin levels in NAFLD patients and control subjects demonstrated an overall MD of 3.361 (95% CI −0.175–6.897). Substantial heterogeneity was reported with an I^2^ = 97.09% and *p*-value < 0.001.

Furthermore, a subgroup analysis was conducted according to the diagnosis method, using liver biopsy and ultrasonography. A total of five studies were included in the pooled analysis involving liver-biopsy-confirmed hepatic steatosis [35,36,44,45,46], with an overall MD of 1.337 (95% CI −1.877–4.551), heterogeneity reported with an I^2^ = 85.20% and *p*-value 0.069. A total of four studies were included in the pooled analysis involving hepatic steatosis evaluated using ultrasonography [41,52,53,55], with an overall MD of 5.013 (95% CI −1.568–11.593), heterogeneity reported with an I^2^ = 97.44%, and *p*-value < 0.001.

### 3.5. Serum Visfatin Levels in NASH vs. Controls

Serum visfatin levels were evaluated in a total of three studies comparing NASH patients with control subjects [36,44,45]. The pooled studies for the analysis assessing serum visfatin levels in NASH patients and control subjects demonstrated an overall MD of −2.038 (95% CI −6.839–2.763). Substantial heterogeneity was reported with an I^2^ = 93.6% and *p*-value = 0.012.

### 3.6. Serum Visfatin Levels in Simple Steatosis vs. Controls

Serum visfatin levels were evaluated in a total of two studies comparing simple steatosis patients with control subjects [36,44]. The pooled studies for the analysis assessing serum visfatin levels in simple steatosis patients and control subjects demonstrated an overall MD of 7.523 (95% CI −16.221–31.267). Moderate heterogeneity was reported with an I^2^ = 56.99% and *p*-value = 0.127.

**Figure 3:**
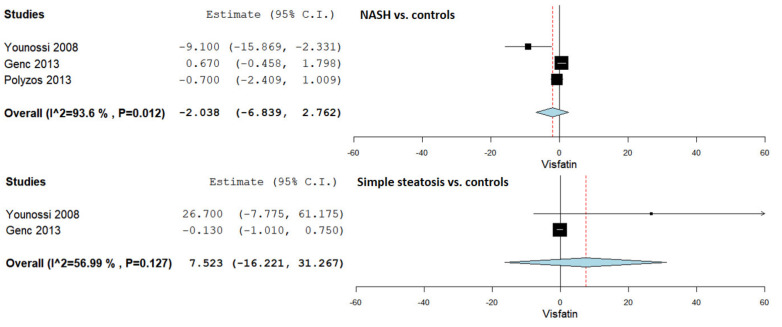
Studies evaluating serum visfatin levels in NASH vs. controls and simple steatosis vs. controls.

Figure 3 summarizes the obtained results evaluating serum visfatin levels in NASH vs. controls and simple steatosis vs. controls.

### 3.7. Serum Visfatin Levels in NASH vs. Simple Steatosis

**Figure 4:**
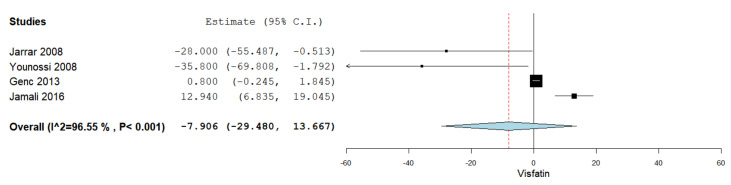
Studies evaluating serum visfatin levels in NASH vs. simple steatosis.

Serum visfatin levels were evaluated in a total of four studies comparing NASH patients with simple steatosis patients [35,36,44,48]. Figure 4 summarizes the obtained meta-analysis results. The pooled studies for the analysis assessing serum visfatin levels in NASH patients and simple steatosis patients demonstrated an overall MD of −7.906 (95% CI −29.480–13.667). Substantial heterogeneity was reported with an I^2^ = 96.55% and *p*-value < 0.001.

### 3.8. Serum Visfatin Levels in Simple Steatosis vs. Moderate Severe Steatosis

**Figure 5:**
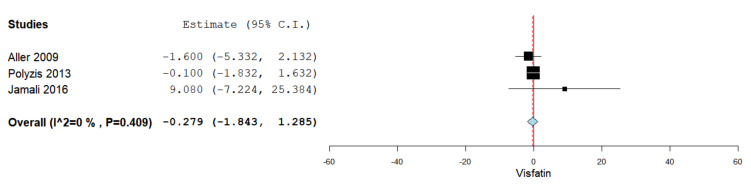
Studies evaluating serum visfatin levels in simple steatosis vs. moderate to severe steatosis.

Serum visfatin levels were evaluated in a total of three studies comparing simple steatosis patients with patients having moderate to severe hepatic steatosis [37,45,47]. Figure 5 summarizes the obtained meta-analysis results. The pooled studies for the analysis assessing serum visfatin levels in simple steatosis patients and moderate to severe steatosis patients demonstrated an overall MD of −0.279 (95% CI −1.843–1.285). No heterogeneity was reported with an I^2^ = 0% and *p*-value = 0.409.

### 3.9. Serum Visfatin Levels in the Presence vs. Absence of Lobar Inflammation

**Figure 6:**
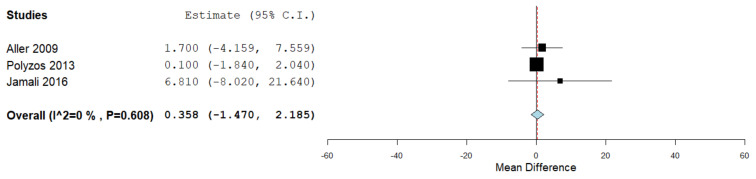
Studies evaluating serum visfatin levels in the presence vs. absence of lobar inflammation.

Serum visfatin levels were evaluated in a total of three studies, comparing the values in the presence and absence of lobar inflammation assessed through liver biopsy and histopathological evaluation [37,45,47]. Figure 6 summarizes the obtained meta-analysis results. The pooled studies for the analysis assessing serum visfatin levels in the presence and absence of lobar inflammation demonstrated an overall MD of 0.358 (95% CI −1.470–2.185). No heterogeneity was reported with an I^2^ = 0% and *p*-value = 0.608.

### 3.10. Serum Visfatin Levels in the Presence vs. Absence of Liver Fibrosis 

**Figure 7:**
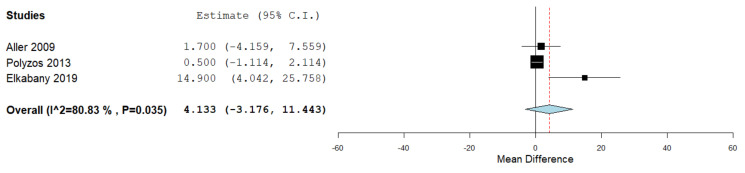
Studies evaluating serum visfatin levels in the presence vs. absence of liver fibrosis.

Serum visfatin levels were evaluated in a total of three studies, comparing the values in the presence and absence of liver fibrosis assessed through liver biopsy and histopathological evaluation in two studies [37,45] and transient elastography in one study [52]. Figure 7 summarizes the obtained meta-analysis results. The pooled studies for the analysis assessing serum visfatin levels in the presence and absence of liver fibrosis demonstrated an overall MD of 4.133 (95% CI −3.176–11.443). Substantial heterogeneity was reported with an I^2^ = 80.83% and *p*-value = 0.035.

### 3.11. Serum Visfatin Levels in Males vs. Females

**Figure 8:**
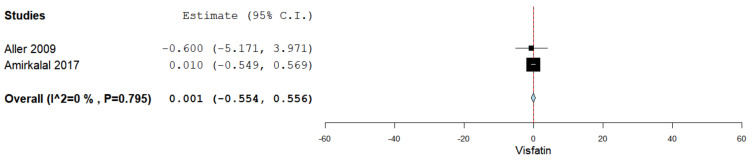
Studies evaluating serum visfatin levels in males vs. females.

Serum visfatin levels were evaluated in a total of two studies comparing the values in males and females [37,45,47]. Figure 8 summarizes the obtained meta-analysis results. The pooled studies for the analysis assessing serum visfatin levels in males and females demonstrated an overall MD of 0.001 (95% CI −0.554–0.556). No heterogeneity was reported with an I^2^ = 0% and *p*-value = 0.795.

### 3.12. Quality Assessment

The NHLBI quality assessment tools were used to evaluate the methodological quality of eligible studies included in the qualitative assessment of our review, as demonstrated in Supplementary Table S2. A total of 10 articles were evaluated using the NHLBI quality assessment of case-control studies [35,36,38,39,43,44,46,47,53,55] and 11 articles using the NHLBI quality assessment tool for observational cohort and cross-sectional studies [37,40,41,42,45,48,49,50,51,52,54].

There were several issues that we found regarding the presence of bias in the evaluated articles. Eleven articles received an overall rating of “fair” [38,39,41,42,44,45,47,49,51,52,53], while five articles were rated as “good” [36,46,48,54,55], and five studies were rated as “poor” [35,37,40,43,50]. In general, all included articles had a clearly stated research question or objective. The study population was clearly specified and defined in 14 studies [36,38,39,40,41,42,43,44,45,46,48,52,54,55]. In almost half (4 out of 10) of the case-control studies, it was not reported, or we could not determine if controls were selected from a similar population that gave rise to the cases [35,38,43,44]. Eight articles out of twenty-one used ultrasonography for the disease diagnostic [41,49,50,51,52,53,54,55], eight of them in the cross-sectional design studies. All the studies used measures of exposure that were clearly defined, valid, reliable, and implemented consistently. Only four articles reported that the assessors of the exposure were blinded to the status of the participants [36,38,41,44,46,48,53]. Only 1 of the 11 cross-sectional studies reported that the outcome assessors were blinded to the exposure status of the participants [41]. Twelve studies assessed potential cofounding variables and performed statistical adjustments for their impact [35,36,37,38,46,47,48,49,50,51,53,55]. None of the cross-sectional studies measured the exposure prior to the outcome, nor was the timeframe between the two sufficient to expect to see an association between the exposure and the outcome if it existed. Also, none of the case-control studies could confirm that the exposure occurred prior to the development of the condition.

## 4. Discussion

Lately, there has been a growing interest in evaluating several adipokines that are possibly associated with NAFLD, including visfatin. Although the current literature contains several published systematic reviews and meta-analyses evaluating adipokines in NAFLD, none evaluated serum visfatin levels in NAFLD [56,57,58]. To the best of our knowledge, this is the first systematic review and meta-analysis to evaluate the association between serum visfatin levels and NAFLD, the presence and severity of hepatic steatosis, as well as liver fibrosis, lobar inflammation, NASH, and gender differences. We included 21 articles in our qualitative synthesis with a total study population of approximately 1900 subjects from different races and backgrounds who participated in 10 cross-sectional studies, 9 case-control studies, and 2 prospective studies that were conducted in Europe, the Middle East, Asia and America. Moreover, we included 14 articles in our quantitative synthesis. We demonstrated that serum visfatin levels are not significantly associated with NAFLD, the presence or severity of hepatic steatosis, liver fibrosis, lobar inflammation, NASH, and gender differences. 

We reported several results that need to be further discussed. Firstly, the term NAFLD was recently updated to metabolic-dysfunction-associated fatty liver disease (MAFLD) with new criteria for diagnosis. MAFLD is characterized by the presence of hepatic steatosis, in addition to one of the following three criteria, including overweight/obesity, type 2 diabetes mellitus (DM), or confirmed metabolic dysregulation [59,60]. Therefore, NAFLD and MAFLD should not be used interchangeably because of the difference in diagnostic criteria between the two terms. The current literature lacks studies evaluating serum visfatin levels in MAFLD. Hence, all studies included in our systematic review and meta-analysis used the diagnostic criteria for NAFLD, and not MAFLD, reflecting findings associated with NAFLD and not MAFLD. Therefore, future studies are required to evaluate serum visfatin levels using the MAFLD criteria. 

Secondly, we reported a prevalence of NAFLD in our sample study of approximately 50%, with an almost equal sex distribution. These findings might be explained by sampling methods used in the included studies. Included studies were from various continents involving participants from several backgrounds. As several risk factors and pathologies have been demonstrated to be associated with specific races and ethnicities, including studies involving subjects from multiple races allows us to report more reliable and generalizable results that are based on findings involving participants from different backgrounds. 

Thirdly, we included studies that used a variety of methods to evaluate the presence and severity of hepatic steatosis. Diagnosing NAFLD can be confirmed by the presence of hepatic steatosis through a liver biopsy (which is the current gold standard), in addition to several other imaging methods, including ultrasonography (which is currently the most commonly used investigation to evaluate hepatic steatosis), as well as CT scans, MRI, and noninvasive biomarkers [1,61,62]. Almost half of the included studies in our review performed a liver biopsy to assess for hepatic steatosis, while the rest of the studies used ultrasonography. We did not include studies that confirmed the diagnosis of NAFLD through the sole use of liver enzymes such as ALT levels [63,64].

Fourthly, in addition to visfatin, several other adipokines have been studied in NAFLD, including leptin, adiponectin, resistin, and chemerin. Current studies reported controversial potential effects of visfatin in regard to insulin resistance, hepatic steatosis, and fibrosis. However, one of the most studied adipokines, adiponectin, was reported to be associated with potential effects leading to an improvement in insulin resistance, as well as hepatic steatosis, inflammation, and fibrosis [56]. Moreover, although leptin was demonstrated to improve insulin resistance and liver fat, it was also reported to deteriorate hepatic inflammation and fibrosis [56].

Fifthly, interestingly, although our findings demonstrated that visfatin is not associated with NAFLD, the presence and severity of hepatic steatosis, liver fibrosis, lobar inflammation, NASH, and gender differences, a couple of recently published studies reported a significant association between visfatin and hepatocellular carcinoma (HCC), suggesting that visfatin plays an essential role in the proliferation of HCC cells and may also be associated with disease progression [65,66,67,68]. Further future studies are required in order to understand the principles and possible mechanisms through which visfatin could possibly lead to an increased risk of HCC without leading to an increased hepatic steatosis severity or inflammation in NAFLD. Understanding how the signaling pathways that potentially play a role in controlling the expansion of adipose tissue and inflammation is considered crucial in order to prevent obesity-associated comorbidities [69].

Sixthly, NAFLD is mainly a hepatic pathology with several extrahepatic manifestations, including cardiovascular complications, which are the main leading cause of death in NAFLD patients, mostly attributed to ischemic heart disease [70,71,72]. Increased visfatin levels were reported in patients with atherosclerotic and coronary artery disease, both diseases demonstrated to be among the main mortality causes in NAFLD [16,17,18,19]. Current literature lacks studies evaluating CVD (mainly atherosclerotic disease) and visfatin levels in NAFLD patients. It remains to be demonstrated in future studies if NAFLD patients who have concomitant atherosclerotic or coronary artery disease will have increased visfatin levels. Emerging evidence points to the existence of several obesity phenotypes being associated with different CV risk factors, suggesting a relation to the physical and lifestyle features [73]. This might explain how CV prognosis might be improved in certain overweight and obese subjects compared to leaner ones, also known as the obesity paradox. Due to the limited number of available studies evaluating serum visfatin levels in portal inflammation, in addition to visceral adipose tissue and liver visfatin levels in NAFLD and liver fibrosis, we were not able to conduct a meta-analysis for further assessment of these associations. 

Seventhly, according to the quality assessment of included studies in our systematic review and meta-analysis, almost half of the studies were rated as “fair”, while five studies were rated as “good” and “poor” each. Hence, results obtained from studies rated as “fair” and “poor” should be cautiously interpreted. As global quality assessment measures are not considered clear enough to identify specific biases in articles, we detailed the description of the items that help with this understanding. Thus, almost half might be subject to selection bias (the population was not clearly specified, or the controls were not selected from a similar population as the cases for sure). The exposure was valid and reliable in all of the studies. Almost half of the studies used ultrasonography for disease diagnostic, instead of liver biopsy, due to associated risk for the latter technique. Ultrasonography is a technique with high specificity but low sensitivity for fatty liver disease diagnostic; thus, directional misclassification might have occurred, which could contribute to the reduction of the relation between the exposure and the outcome (a bias towards the null) [74,75]. Although few studies used blinding, due to the objective measured used, this could not negatively impact the study findings. Another important negative issue is the fact that almost half of the studies did not control for confounding. Last, due to the study design used, where the timeframe between the exposure and the outcome measure was short and in the absence of the possibility to establish precedence between the two, we cannot know which was first, the exposure of the disease or the outcome. 

Our systematic review and meta-analysis has several limitations. Due to the observational design of the included studies in this review, causality between visfatin and NAFLD, hepatic steatosis, liver fibrosis, lobar inflammation, and NASH cannot be confirmed or negated. Although almost half of the included studies used liver biopsy, which is the current gold standard to diagnose NAFLD, the remaining half used ultrasonography, which might possibly lead to underestimation in NAFLD prevalence. Almost half of the studies might be at some risk of selection bias. Also, nearly half of the studies did not control for confounding such as pharmacological treatment or associated comorbidities, which affect metabolic pathways and potentially confound visfatin synthesis, and even for them, residual confounding might exist due to the observational nature of the studies. Moreover, there is heterogeneity among studies with respect to BMI, where adipose tissue may have a significant impact on visfatin levels. However, due to incomplete characteristics of patients in analyzed studies, we were not able to perform further detailed subgroup analysis. Furthermore, due to the limited number of published studies evaluating visfatin levels in NASH, liver fibrosis, lobar inflammation, and hepatic steatosis severity, we were able to assess only a few studies, about two or three studies for each association. Therefore, further studies evaluating these associations are considered necessary. Results should be interpreted with caution due to possible methodological flaws in included studies.

Nevertheless, our systematic review and meta-analysis also has several important strengths. The topic of this review is of important clinical significance, mainly due to the rapid global increase in the prevalence of NAFLD, as well as the associated increased morbidity and mortality rates. We believe that our review points out the missing required data that requires further assessment in future studies while summarizing the current literature in a nonbiased manner. Moreover, we conducted the search strategy in a comprehensive manner using several medical databases, which allowed us to assess the studied association in a systematic manner. We included studies involving participants from several races and backgrounds, which allowed us to have more generalizable results. To the best of our knowledge, this is the first systematic review and meta-analysis to evaluate the association between visfatin levels and NAFLD, hepatic steatosis presence and severity, liver fibrosis, lobar inflammation, NASH, and gender differences.

## 5. Conclusions and Future Directions

In conclusion, we could not find evidence to sustain that visfatin levels are associated with NAFLD, the presence or severity of hepatic steatosis, liver fibrosis, lobar inflammation, NASH, and gender differences. Nevertheless, obtained results should be interpreted with caution due to the imperfect methodological quality of the assessed studies.

Future research is required in order to evaluate serum visfatin levels in the newly defined MAFLD using the new diagnosis criteria, as well as in patients with portal inflammation and NAFLD patients with concomitant atherosclerotic cardiovascular disease. Moreover, current studies evaluating visfatin levels in visceral adipose tissue and liver in NAFLD and liver fibrosis are very limited, requiring future research for further evaluation. Furthermore, possible mechanisms that might associate elevated visfatin levels with HCC remain to be investigated.

## Supplementary Materials

The following are available online at https://www.mdpi.com/article/10.3390/jcm10143029/s1, Complementary Material: Search Strategy; Table S1: Studies evaluating visfatin levels in NAFLD, Table S2: NHLBI Quality Assessment of Case-Control Studies.
