# Supplementary material for: Serum Visfatin Levels in Nonalcoholic Fatty Liver Disease and Liver Fibrosis: Systematic Review and Meta-Analysis
Source: J Clin Med. 2021 Jul 7;10(14):3029. doi: 10.3390/jcm10143029 (PMC8306785; doi:10.3390/jcm10143029)
Supplement: Supplementary file 1 [file jcm-10-03029-s001.zip › Supplementary Materials.pdf]

Supplementary Material 1

Search Strategy:

We used the following search string for PubMed: ((“Non alcoholic Fatty Liver Disease”) OR (“NAFLD”) OR (“Nonalcoholic Fatty Liver Disease”) OR (“Non-alcoholic Fatty Liver Disease”) OR (“Nonalcoholic fatty liver”) OR (“Nonalcoholic fatty livers”) OR (“Nonalcoholic Steatohepatitis”) OR (“Nonalcoholic Steatohepatitides”) OR (NASH)) AND ((Nicotinamide Phosphoribosyltransferase) OR (NAMPT Protein) OR (“Pre-B-Cell Colony-Enhancing Factor”) OR (“Pre B Cell Colony Enhancing Factor”) OR (Visfatin) OR (NAmPRTase) OR (NMN Pyrophosphorylase)) and a similar one for EMBASE and Cochrane Library.

Extracted data included author names, publication year, country, study design as well as study characteristics including total subjects, mean age, gender, NAFLD diagnosis, NAFLD percentage, visfatin levels in serum, liver or adipose tissue, in addition to the main study findings that were entered into tables, while final data was collated and presented in the manuscript text.
