# Supplementary material for: Serum Visfatin Levels in Nonalcoholic Fatty Liver Disease and Liver Fibrosis: Systematic Review and Meta-Analysis
Source: J Clin Med. 2021 Jul 7;10(14):3029. doi: 10.3390/jcm10143029 (PMC8306785; doi:10.3390/jcm10143029)
Supplement: Supplementary file 1 [file jcm-10-03029-s001.zip › Supplementary Tables S1 and S2.pdf]

Supplementary Table S1. Studies evaluating visfatin levels in NAFLD

First Author / Year / Country	Study Design	Study Characteristics	Main Findings
**Jarrar MH et al. / 2008 / USA** [39]	Case-control	• **Total Subjects:** 95 (NASH – 26; SS – 19; Obese Controls – 38; Non-obese Controls – 12)<br>• **NAFLD:** 45 (47.37%)<br>• **Mean age (years):** SS 37 ± 9.2; NASH 43.9 ± 11.4; Obese Controls 40 ± 9.5; Non-obese Controls 56 ± 17<br>• **NAFLD diagnosis:** Liver biopsy<br>• **Gender (males):** 24 (25.26%)<br>• **Serum Visfatin Levels (pg/mL):** NAFLD 28.9 ± 41.6; Obese Controls 26.8 ± 19.0; (p value= 0.40; adjusted p value= 0.40).<br>NASH 17.1 ± 6.2; Simple Steatosis 45.1 ± 60.9; (p value= 0.03; adjusted p value= 0.06).	Serum visfatin levels were higher in all obese groups compared to non-obese controls. Visfatin levels were lower in patients with NASH compared to SS and obese controls. In NASH, visfatin was not an independent predictor of histological fibrosis stage.
**Younossi ZM et al. / 2008 / USA** [40]	Case-control	• **Total Subjects:** 101 (NASH – 22; SS – 15; Matched Controls – 32; Validation Set – 32 out of which 21 with NASH and 11 with SS)<br>• **NAFLD:** 69 (68.32%)<br>• **Mean age (years):** SS 37.4 ± 8.3; NASH 42.5 ± 10.4; Matched Controls 39.3 ± 9.8; Validation Set 41.6 ± 10.6<br>• **NAFLD diagnosis:** Liver biopsy<br>• **Gender (males):** 46 (45.55%)<br>• **Serum Visfatin Levels (pg/mL):** SS 52.5 ± 67.0; NASH 16.7 ± 6.3; Matched Controls 25.8 ± 18.0; (p<0.008)	Lower serum visfatin levels were reported in NASH patients.
**Aller R et al. / 2009 / Spain** [41]	Cross-sectional	• **Total Subjects:** 55 (NAFLD – 55)<br>• **NAFLD:** 55 (100%)<br>• **Mean age (years):** 42.8 ± 11.2<br>• **NAFLD diagnosis:** Liver biopsy<br>• **Gender (males):** 37 (67.27%)<br>• **Serum Visfatin Levels (ng/mL):** Low Grade Steatosis 14.1 ± 6.6; High Grade Steatosis 15.7 ± 7.3; (p value= 0.78)<br>Females 14.3 ± 8.9; Males 14.9 ± 6.2; (p value= 0.31)	Concentrations of serum visfatin were reported to be correlated with an increased portal inflammation 1.22 (CI 95%:1.02–1.48) with each 1 ng/ml of visfatin concentration, even after performing logistic regression with age, sex, fat mass and insulin-adjusted portal inflammation, a high grade of steatosis, fibrosis and lobular inflammation as dependent variables.
**Dahl TB et al. / 2010 / Norway** [42]	Case-control	• **Total Subjects:** 85 (NAFLD – 58 out of which 32 with NASH and 26 with SS; Healthy Controls – 27)<br>• **NAFLD:** 58 (68.24%)<br>• **Mean age (years):** –<br>• **NAFLD diagnosis:** Liver biopsy<br>• **Gender (males):** 46 (45.55%)<br>• **Serum Visfatin Levels (pg/mL):** serum levels of NAMPT/visfatin were significantly decreased in patients with NAFLD (n = 58) compared with healthy controls (n = 27), with no differences between NASH (n = 32) and simple steatosis (n = 26) or between different stages of fibrosis (data not shown).<br>• **Hepatic Expression of Visfatin:** NAFLD patients had lower hepatic mRNA levels of NAMPT/visfatin as compared with controls. However, no difference between simple steatosis (n = 6) and NASH (n = 7) was demonstrated.	NAFLD is a significant predictor of serum NAMPT/visfatin levels, independent of all measured metabolic parameters, age, sex, BMI and ALT.
**Gaddipati R et al. / 2010 / India** [43]	Case-control	• **Total Subjects:** 115 (NAFLD – 77 out of which 35 with SS, 30 with moderate steatosis and 12 with NASH; Non-NAFLD – 38)<br>• **NAFLD:** 77 (66.96%)<br>• **Mean age (years):** SS 48.94 ± 13.12; Moderate Steatosis 46.2 ± 12.22; NASH 49 ± 13.19; Non-NAFLD 46.5 ± 16.48<br>• **NAFLD diagnosis:** Liver biopsy<br>• **Gender (males):** 63 (54.78%)<br>• **Visceral Adipose Tissue Visfatin Levels (ng/mg pro):** SS 87.25 ± 75.22 (p <0.05); Moderate Steatosis 61.36 ± 54.46 (p<0.001); NASH 31.83 ± 29.43 (p<0.001); Non-NAFLD 210.38 ± 93.16.	Patients with NAFLD presented with a significant reduction in VAT visfatin level that was associated with the steatosis severity.
**Kukla M et al. / 2010 / Poland** [44]	Cross-sectional	• **Total Subjects:** 40 (NAFLD – 40)<br>• **NAFLD:** 40 (100%)<br>• **Mean age (years):** 42.2 ± 9.1	Morbidly obese patients with NAFLD and marked liver fibrosis were associated with increased hepatic visfatin expression that was positively associated with

		• **NAFLD diagnosis:** Liver biopsy<br>• **Gender (males):** 16 (40%)<br>• **Hepatic Expression of Visfatin:** Mean value 1.00 ± 0.66; Visfatin expression significantly increased among the patients with fibrosis vs. without fibrosis, 1.09 ± 0.65 vs. 0.36 ± 0.03; respectively; p = 0.036. A comparison of patients with F1 (including stage 1A, 1B, 1C) to those with bridging fibrosis/cirrhosis (F3-F4) demonstrated a more pronounced visfatin expression in patients with more advanced fibrosis but not reaching statistical significance – 0.88 ±0.66 vs. 1.23 ±0.68; p = 0.24. The fibrosis stage and visfatin expression in the liver tissue were statistically significant with a positive correlation (r = 0.52, p = 0.03).<br>However, no significant association was demonstrated between visfatin tissue expression with either the grade of lobular and portal inflammation or the grade of steatosis (respectively: r = 0.01, p = 0.95; r = 0.16, p = 0.51; r = –0.35, p = 0.34). Moreover, no statistical significance between patients with NASH and simple steatosis with visfatin expression (1.11 ±0.71 vs. 0.62 ±0.19, p = 0.54). Furthermore, NAS score was not related to visfatin expression (r = –0.26, p = 0.31) with no differences in visfatin expression index value among patients with NAS 0-2 vs. 3-4 vs. 5-8 (1.05 ±0.56 vs. 0.76 ±0.60 vs. 1.06 ±0.80, p = 0.58, respectively).	fibrosis stage suggesting a potential role of visfatin in the pathogenesis and progression of fibrosis in NAFLD patients. However, no significant association was reported regarding hepatic steatosis and inflammation.
**Akbal E et al. / 2012 / Turkey** [45]	Prospective cohort	• **Total Subjects:** 57 (NAFLD – 30; Healthy Controls – 27)<br>• **NAFLD:** 30 (52.63%)<br>• **Mean age (years):** NAFLD 41.1 ± 9.1; Controls 43.6 ± 10.2<br>• **NAFLD diagnosis:** Ultrasonography<br>• **Gender (males):** 29 (50.88%)<br>• **Serum Visfatin Levels (ng/mL):** NAFLD 14.7 ± 8.1; Healthy Controls 9.4 ± 1.6; (p < 0.001)	Significantly elevated serum visfatin levels were reported in NAFLD patients compared with healthy controls. Moreover, no relationship between serum visfatin levels with HOMA-IR, insulin levels, and BMI was demonstrated
**Yoon MY et al. / 2012 / Korea** [46]	Cross-sectional	• **Total Subjects:** 30 (NASH – 10; Non-NASH – 20)<br>• **NAFLD:** 30 (100%)<br>• **Mean age (years):** NASH 42.5 (37.75-52.75); Non-NASH 52.5 (38.25-65.00)<br>• **NAFLD diagnosis:** Liver biopsy<br>• **Gender (males):** 30 (100%)<br>• **Serum Visfatin Levels (ng/mL):** NASH 8 (3.15 - 16.96); Non-NASH 6.29 (2.6 - 11.48); (p value= 0.451)<br>• **Tissue Expression of Visfatin:**<br>**PCR –**<br>**Liver:** Non-NASH 89.36 (84.17-99.37); NASH 87.59 (68.76-92.19); (p value= 0.328)<br>**Visceral FAT:** Non-NASH 88.69 (83.32-98); NASH 95.8 (83.41-106.81); (p value= 0.502)<br>**SUQ FAT:** Non-NASH 103.67 (93.1-119.46); NASH 103.14 (87.5-117.11); (p value= 0.660)<br>**Western –**<br>**Liver:** Non-NASH 95.82 (61.65-114.08); NASH 73.55 (48.75-109.87); (p value= 0.475)<br>**Visceral FAT:** Non-NASH 53.66 (28.43-85.99); NASH 33.46 (21.13-62.99); (p value= 0.113)<br>**SUQ FAT:** Non-NASH 99.5 (69.08-465.17); NASH 71.74 (52.04-493.77); (p value= 0.202)	Patients with NASH vs. non-NASH did not present significant differences in visfatin levels.
**Auguet T et al. 2013 / Spain** [47]	Case-control	• **Total Subjects:** 133 (Morbidly Obese – 95; Controls – 38)<br>• **NAFLD:** 69 (51.88%)<br>• **Mean age (years):** Controls 45.4 ± 16.3; Morbidly Obese 46.8 ± 10.6<br>• **NAFLD diagnosis:** Liver biopsy (performed in 88 Morbidly obese subjects and 5 normal weight controls)<br>• **Gender (males):** 0 (0%)<br>• **Serum Visfatin Levels (ng/mL):**<br>Controls 1.4 ± 0.9; Morbidly Obese 3.3 ± 2.6; (p <0.001).<br>Morbidly Obese NAFLD non-diabetic 3.1 ± 2.2; Morbidly Obese NAFLD diabetic 4.3 ± 3.2; (p value= ns)<br>Morbidly Obese Non-diabetics 2.8 ± 2.0; Morbidly Obese Diabetics 4.2 ± 3.1; (p value= 0.016)<br>• **Hepatic Expression of Visfatin:** Visfatin liver expression was significantly higher in morbidly obese women with NAFLD than in morbidly obese women with normal liver (data not shown).	Morbidly obese females presented with elevated circulating visfatin levels and liver expression compared to healthy controls. This finding was more pronounced in morbidly obese NAFLD patients compared to those with a normal liver histology. Serum visfatin levels and hepatic expression of visfatin correlate positively with pro-inflammatory adipocytokines including IL-6, resistin, TNF-α, and CRP.

**Genc H et al. / 2013 / Turkey** [48]	Case-control	• **Total Subjects:** 174 (NAFLD – 114; Controls – 60)<br>• **NAFLD:** 114 (65.52%)<br>• **Mean age (years):** NAFLD 32.00 (20.00-45.00); Controls 29.00 (21.00-43.00<br>• **NAFLD diagnosis:** Liver biopsy<br>• **Gender (males):** 174 (100%)<br>• **Serum Visfatin Levels (ng/mL):** NAFLD 13.66 ± 2.35; Controls 13.33 ± 2.73; (p value= 0.416)<br>Controls 13.33 ± 2.73; NASH 14.00 (8.60-21.20); SS 13.2 (10.80-17.00); Borderline NASH 14.30 (9.20-19.10); Controls vs. NASH (p value= 0.162); Controls vs. SS (p value= 0.402); Controls vs. Borderline NASH (p value= 0.307)	Plasma visfatin levels were not modified in early stages of NAFLD, while being inversely associated with TNF-α suggesting a role for visfatin in protection against liver injury. No significant relationship between visfatin with steatosis grade, ballooning degeneration grade, lobar inflammation grade and fibrosis stage was reported.
**Polyzos AS et al. / 2013 / Greece** [49]	Cross-sectional	• **Total Subjects:** 54 (NAFLD – 30 out of which 15 with NAFL and 15 with NASH; Controls – 24)<br>• **NAFLD:** 30 (55.56%)<br>• **Mean age (years):** Controls 56 (52-61); NAFL 55 (44-60); NASH 54 (50-63)<br>• **NAFLD diagnosis:** Liver biopsy<br>• **Gender (males):** 12 (22.22%)<br>• **Serum Visfatin Levels (ng/mL):** Controls 6.4 (3.9-7.6); NAFL 5.3 (4.2-6.6); NASH 5.7 (4.2-7.7); (p value= 0.986)	No significant differences in visfatin within the lesions of steatosis grade, fibrosis stage, ballooning, lobular and portal inflammation with similar serum visfatin levels between NAFL and NASH patients.
**Jamali R et al. / 2016 / Iran** [50]	Case-control	• **Total Subjects:** 108 (NAFLD – 54; Controls – 54)<br>• **NAFLD:** 54 (50%)<br>• **Mean age (years):** NAFLD 37.02 ± 9.82; Controls 33.24 ± 12.02<br>• **NAFLD diagnosis:** Liver biopsy<br>• **Gender (males):** 57 (52.78%)<br>• **Serum Visfatin Levels (ng/mL):** NAFLD 19.96 ± 17.5; Controls 12.68 ± 13.21; (significant difference between groups)<br>***- Independent Predictors of Nonalcoholic Fatty Liver Disease***<br>Visfatin – Regression Coefficient (beta) 1.05; (95% CI 1.001–1.09); (p value= 0.04)<br>***- Independent Predictors of Nonalcoholic Steatohepatitis***<br>Visfatin – Regression Coefficient (beta) 1.05; (95% CI 1.007–1.09); (p value= 0.02)<br>***- Best Threshold Values of Biomarkers for Differentiating Nonalcoholic Fatty Liver Disease Patients From Healthy Subjects According to ROC Analysis***<br>Visfatin (ng/mL) – Serum concentration 6.35; Sensitivity 74%, Specificity 50%; AOC (95% CI): 0.65 (0.53–0.76)	Increased levels of serum visfatin were independently associated with an increased likelihood of NAFLD presence evaluated using binary logistic regression. Furthermore, an increased probability of NASH presence was associated with increased circulating visfatin levels.
**Jamali R et al. / 2016 / Iran** [51]	Case-control	• **Total Subjects:** 36 (NAFLD – 18; Healthy Controls – 18)<br>• **NAFLD:** 18 (50%)<br>• **Mean age (years):** NAFLD 34.50 ± 8.85; Controls 30.44 ± 10.11<br>• **NAFLD diagnosis:** Liver biopsy<br>• **Gender (males):** 26 (72.22%)<br>• **Serum Visfatin Levels (ng/mL):** NAFLD 20.67 ± 15.96; Healthy Controls 12.45 ± 13.42; (p value= 0.10)<br>***Comparison (Mean ± SD) of Clinical and Laboratory Findings Among Cases with Less or More Than 33% Steatosis, Lobular Inflammation of Grade One or More, and Mild and More Severe Fibrosis***<br>- Steatosis Severity – ≤33%: 27.10 ± 13.24, ≥33%: 18.02 ± 16.69, (p value= 0.30);<br>- Lobar Inflammation Severity – Grade 2-3: 24.07 ± 14.36, Grade 0-1: 17.26 ± 17.58, (p value= 0.38)<br>- Fibrosis Severity – Moderate or Severe: 16.57 ± 14.916, Mild: 23.28 ± 16.72, (p value= 0.40)<br>***Correlation Between the Severity of Steatosis, Lobular Inflammation, and Fibrosis Among the Clinical and Laboratory Findings in NAFLD Patients***<br>- Steatosis Severity – Correlation Coefficient -0.01; CI (-0.08, 0.04); (p value= 0.37)<br>- Lobar Inflammation Severity – Correlation Coefficient -0.01; CI (-0.07, 0.06); (p value= 0.80)<br>- Fibrosis Severity – Correlation Coefficient -0.01; CI (-0.05, 0.04); (p value= 0.65)	No relationship was demonstrated between serum levels of visfatin and steatosis severity, lobar inflammation severity and severity of liver fibrosis with no significant difference in visfatin levels between NAFLD and controls.
**Jamali R et al. / 2016 / Iran** [52]	Cross-sectional	• **Total Subjects:** 54 (NAFLD – 54)<br>• **NAFLD:** 54 (100%)<br>• **Mean age (years):** NAFLD 37.02 ± 9.82<br>• **NAFLD diagnosis:** Liver biopsy	Elevated serum levels of visfatin were independently associated with steatosis grade of > 33%. However, visfatin was not significantly associated with lobar inflammation grade, fibrosis stage and NAS.

		• **Gender (males):** 35 (64.8%)<br>• **Serum Visfatin Levels (ng/mL):** Total 19.96 ± 17.5; Simple Fatty Liver 5.40 ± 0.84; NASH 18.34 ± 16.18<br>***Association between histological findings and serum visfatin levels***<br>Steatosis Degree – OR 1.08; (95% CI 1.030-1.14); (p value= 0.001)<br>***Best cut-off values of serum adipokine levels to differentiate histological groups according to receiver operating characteristic analysis***<br>Visfatin (ng/mL) – Serum concentration: 13.00; Sensitivity 84%; Specificity 69%	
**Amirkalali B et al. / 2017 / Iran** [53]	Cross-sectional	• **Total Subjects:** 62 (NAFLD – 62)<br>• **NAFLD:** 62 (100%)<br>• **Mean age (years):** Males 39.84 ± 12.10; Females 47.83 ± 10.62<br>• **NAFLD diagnosis:** Ultrasonography for hepatic steatosis and Fibroscan for liver stiffness<br>• **Gender (males):** 32 (51.61%)<br>• **Serum NAMPT Levels (ng/mL):** Males 2.44 ± 1.07; Females 2.45 ± 1.17; (p value= 0.98)	Visfatin levels were not significantly different between males and females with NAFLD.
**Amirkalali B et al. / 2017 / Iran** [54]	Cross-sectional	• **Total Subjects:** 62 (NAFLD – 62)<br>• **NAFLD:** 62 (100%)<br>• **Mean age (years):** Males 35.5 (29 – 52); Females 51 (42.75 – 55); Total 46.50 (32.75 – 53.00)<br>• **NAFLD diagnosis:** Ultrasonography for hepatic steatosis and Fibroscan for liver stiffness<br>• **Gender (males):** 32 (51.61%)<br>• **Serum NAMPT Levels (ng/mL):** Males 2.44 ± 1.03; Females 2.45 ± 1.13; Total 2.47 (1.52 – 3.29); (p value= 0.98)	Elevated serum NAMPT in females was associated with a lower hepatic de novo lipogenesis index. However, it was associated with higher hepatic fat in males evaluated in multiple linear regression ($\beta = 0.35$, p = 0.035), which was not significant in univariate linear regression ($\beta = 0.33$, p = 0.07), without an association with the DNL index. These findings suggest a sex dependent association of serum NAMPT in NAFLD prognosis.
**Amirkalali B et al. / 2017 / Iran** [55]	Cross-sectional	• **Total Subjects:** 62 (NAFLD – 62)<br>• **NAFLD:** 62 (100%)<br>• **Mean age (years):** Males 39.84 ± 12.10; Females 47.83 ± 10.62<br>• **NAFLD diagnosis:** Ultrasonography for hepatic steatosis and Fibroscan for liver stiffness<br>• **Gender (males):** 32 (51.61%)<br>• **Serum NAMPT Levels (ng/mL):** Males 2.44 ± 1.07; Females 2.45 ± 1.17; (p value= 0.98)<br>In males, serum visfatin had a significant positive association with serum Aspartate Aminotransferase (AST) (B = 0.47, P = 0.009), alanine aminotransferase (ALT) (B = 0.40, P = 0.035), CK18 (B = 0.50, P = 0.008), and cCK18 (B = 0.47, P = 0.012). In females, serum visfatin only had a weak association with CK18 (B = 0.37, P = 0.045).	In males, elevated serum visfatin levels could be an indicator for more hepatic injury, which is not the case in females suggesting that the contrary findings between serum visfatin and pathologic findings of NAFLD in various studies could be explained could be partly due to different gender distribution.
**Mousavi Z et al. / 2017 / Iran** [56]	Cross-sectional	• **Total Subjects:** 120 patients with MetS (NAFLD – 50; No NAFLD – 70)<br>• **NAFLD:** 50 (41.67%)<br>• **Mean age (years):** NAFLD 47.6 ± 12.7; No NAFLD 46.1 ± 12.1<br>• **NAFLD diagnosis:** Ultrasonography<br>• **Gender (males):** 44 (36.67%)<br>• **Serum NAMPT Levels (ng/mL):** No NAFLD 37.1 ± 1.7; NAFLD 44.4±1.5; (p value= 0.02)	Patients with MetS and NAFLD presented with significantly increased visfatin levels compared to MetS patients without NAFLD. Moreover, visfatin levels were found to be significantly correlated with the degree of fatty liver according to sonographic classification (r= 0.2, p= 0.02).
**Elkabany ZA et al. / 2019 / Egypt** [57]	Cross-sectional Case-control	• **Total Subjects:** 80 obese children and adolescents (NAFLD – 31; No NAFLD – 49)<br>• **NAFLD:** 31 (38.76%)<br>• **Mean age (years):** All patients 9.0 ± 3.1; NAFLD 9.98 ± 3.39; No NAFLD 8.3 ± 2.73<br>• **NAFLD diagnosis:** Ultrasonography for hepatic steatosis and Fibroscan for liver stiffness<br>• **Gender (males):** 42 (52.5%)<br>• **Serum Visfatin Levels (ng/mL):** All patients 27.5 (13–42); NAFLD 32 (25–50); No NAFLD 18 (7.5–40); (p value <0.001)<br>***Abdominal ultrasound – NAFLD:***<br>Present: 32 (25–50); Absent: 18 (7.5–40); (p value <0.001)<br>***NAFLD and ALT > 40 IU/L:***<br>Present: 45 (25–50); Absent: 25 (13–40); (p value= 0.023)	Obese children and adolescents presented with significantly elevated serum visfatin levels compared with non-obese controls. Higher visfatin levels were reported in patients with dyslipidemia, NAFLD, elevated ALT and steatosis defined by CAP. Moreover, serum visfatin was related to the severity of hepatic fibrosis and steatosis. Furthermore, serum visfatin was positively associated with BMI, waist circumference, waist/hip ratio, ALT, total cholesterol, liver stiffness and CAP.

		Transient elastography – Fibrosis stage by livers stiffness:<br>F0: 21.5 (7.5–32); F1: 27.5 (21.5–36); F2–F3: 50 (18–55); (p value= 0.003)<br>***Steatosis stage by CAP:***<br>S0: 19 (7.5–31); S1: 27.5 (18–36.4); S2: 44.7 (18–50); S3: 50 (31.5–60); (p value= 0.002)	
**Johannsen K et al. / 2019 / Germany [58]**	Prospective population-based cohort	• **Total Subjects:** 403 (Year 2002 – Baseline: NAFLD – 221; No NAFLD – 182); (Year 2013: NAFLD – 207; No NAFLD – 196)<br>• **NAFLD:** 2002 – 221 (54.84%); 2013 – 207 (51.36%)<br>• **Mean age (years):** 2002 – 47.46 ± 11.38; 2013 – 58.14 ± 11.36<br>• **NAFLD diagnosis:** Ultrasonography<br>• **Gender (males):** 212 (52.61%)<br>• **Serum Visfatin Levels (ng/mL):**<br>Baseline Year 2002:<br>No NAFLD: 2.63 ± 1.80; Grade I: 2.70 ± 1.90; Grade II/III: 2.46 ± 1.30; (p value= 0.9966)<br>Follow-up Year 2013:<br>No NAFLD: 3.62 ± 3.84; Garde I: 4.02 ± 3.51; Grade II/III: 5.19 ± 4.76; (p value< 0.0001)<br><br>Males Baseline Year 2002:<br>No NAFLD: 2.05 ± 1.15; Grade I: 2.51 ± 2.13; Grade II/III: 2.28 ± 1.32; (p value= 0.5583)<br>Males Follow-up Year 2013:<br>No NAFLD: 3.36 ± 4.23; Grade I: 3.44 ± 2.15; Grade II/III: 5.07 ± 5.71; (p value= 0.0008)<br><br>Females Baseline Year 2002:<br>No NAFLD: 3.09 ± 2.08; Grade I: 2.88 ± 1.61; Grade II/III: 2.79 ± 1.23; (p value= 0.9521)<br>Females Follow-up 2013:<br>No NAFLD: 3.89 ± 3.37; Grade I: 4.70 ± 4.57; Grade II/III: 5.33 ± 3.45; (p value= 0.0158)	Baseline results showed no significant association between visfatin levels and NAFLD. However, during follow-up, visfatin levels were weakly associated in univariate analysis with NAFLD. This association lost its significance in the partial correlation when taking BMI, waist/hip ratio, and age into account.
**Qiu Y et al. / 2019 / China [59]**	Case-control	• **Total Subjects:** 211 (NAFLD –100; Controls – 111)<br>• **NAFLD:** 100 (47.39%)<br>• **Mean age (years):** Total 33.18 ± 6.15; NAFLD 33.09 ± 5.57; Controls 33.25 ± 6.66<br>• **NAFLD diagnosis:** Ultrasonography<br>• **Gender (males):** 177 (83.89%)<br>• **Serum Visfatin Levels (ng/mL):** Total: 28.46 (24.08–35.62); NAFLD: 26.94 (23.30–33.13); Controls: 30.51 (25.08–38.47); (p value= 0.016)	NAFLD patients presented lower levels of visfatin compared to controls. Using multivariate logistic analysis, circulating visfatin levels were inversely associated with the risk of NAFLD (all p-trend < 0.05) with an OR of 0.30 (95% CI 0.10–0.91) indicating that lower levels of circulating visfatin were independently associated with an increased risk of NAFLD.

ALT – Alanine aminotransferase; BMI – Body mass index; CAP – Controlled attenuation parameter; CRP – C-reactive protein; HOMA-IR – Homeostatic Model Assessment of Insulin Resistance; IL-6 – Interleukin 6; NAFL – Nonalcoholic fatty liver; NAFLD – Nonalcoholic fatty liver disease; NAMPT – Nicotinamide phosphoribosyltransferase; NAS – NAFLD Activity Score NASH – Nonalcoholic steatohepatitis; PCR – Polymerase chain reaction; SUQ – Subcutaneous fat tissue; RNA – Ribonucleic acid; ROC – Receiver Operating Characteristic; SS – Simple steatosis; TNF-α – Tumor necrosis factor alpha; VAT – Visceral adipose tissue.

Supplementary Table S2. NHLBI Quality Assessment of Case-Control Studies

**Criteria**	Jarrar MH et al.[39]	Younossi ZM et al.[40]	Dahl TB et al.[42]	Gaddipati R et al.[43]	Auguet T et al.[47]	Genc H et al.[48]	Jamali R et al.[50]	Jamali R et al.[51]	Elkabany ZA et al.[57]	Qiu Y et al.[59]
1. Was the research question or objective in this paper clearly stated and appropriate?	Yes	Yes	Yes	Yes	Yes	Yes	Yes	Yes	Yes	Yes
2. Was the study population clearly specified and defined?	No	Yes	Yes	Yes	Yes	Yes	Yes	No	No	Yes
3. Did the authors include a sample size justification?	No	No	No	No	No	No	No	No	No	Yes
4. Were controls selected or recruited from the same or similar population that gave rise to the cases (including the same timeframe)?	NR	Yes	NR	Yes	NR	CD	Yes	Yes	CD	Yes
5. Were the definitions, inclusion and exclusion criteria, algorithms or processes used to identify or select cases and controls valid, reliable, and implemented consistently across all study participants?	Yes	Yes	Yes	Yes	Yes	Yes	Yes	Yes	Yes	Yes
6. Were the cases clearly defined and differentiated from controls?	Yes	Yes	Yes	Yes	Yes	Yes	Yes	Yes	Yes	Yes
7. If less than 100 percent of eligible cases and/or controls were selected for the study, were the cases and/or controls randomly selected from those eligible?	NA	NA	NA	NA	NA	NA	NA	NA	NA	NA
8. Was there use of concurrent controls?	CD	Yes	No	Yes	CD	No	Yes	CD	CD	CD
9. Were the investigators able to confirm that the exposure/risk occurred prior to the development of the condition or event that defined a participant as a case?	No	No	No	No	No	No	No	No	No	No
10. Were the measures of exposure/risk clearly defined, valid, reliable, and implemented consistently (including the same time period) across all study participants?	Yes	Yes	Yes	Yes	Yes	Yes	Yes	Yes	Yes	Yes
11. Were the assessors of exposure/risk blinded to the case or control status of participants?	NR	NR	Yes	NR	NR	NR	Yes	NR	NR	NR
12. Were key potential confounding variables measured and adjusted statistically in the analyses? If matching was used, did the investigators account for matching during study analysis?	Yes	Yes	Yes	No	No	No	Yes	Yes	Yes	Yes
Rating	Poor	Good	Fair	Fair	Poor	Fair	Good	Fair	Fair	Good

Supplementary Table S2 (cont'd). NHLBI Quality Assessment Tool for Observational Cohort and Cross-Sectional Studies

**Criteria**	Aller R et al.[41]	Kukla M et al.[44]	Akbal E et al.[45]	Yoon MY et al.[46]	Polyzos SA et al.[49]	Jamali R et al.[52]	Amirkalali B et al.[53]	Amirkalali B et al.[54]	Amirkalali B et al.[55]	Mousavi Z et al.[56]	Johannsen K et al.[58]
1. Was the research question or objective in this paper clearly stated?	Yes	Yes	Yes	Yes	Yes	Yes	Yes	Yes	Yes	Yes	Yes
2. Was the study population clearly specified and defined?	No	Yes	Yes	Yes	Yes	Yes	No	No	No	Yes	Yes
3. Was the participation rate of eligible persons at least 50%?	CD	CD	CD	CD	CD	CD	CD	CD	CD	CD	Yes
4. Were all the subjects selected or recruited from the same or similar populations (including the same time period)? Were inclusion and exclusion criteria for being in the study prespecified and applied uniformly to all participants?	CD	CD	Yes	Yes	Yes	Yes	Yes	Yes	Yes	Yes	Yes
5. Was a sample size justification, power description, or variance and effect estimates provided?	No	No	No	No	No	Yes	Yes	No	Yes	No	Yes
6. For the analyses in this paper, were the exposure(s) of interest measured prior to the outcome(s) being measured?	No	No	No	No	No	No	No	No	No	No	Yes
7. Was the time frame sufficient so that one could reasonably expect to see an association between exposure and outcome if it existed?	No	No	No	No	No	No	No	No	No	No	Yes
8. For exposures that can vary in amount or level, did the study examine different levels of the exposure as related to the outcome (e.g., categories of exposure, or exposure measured as continuous variable)?	Yes	Yes	Yes	Yes	Yes	Yes	No	No	Yes	Yes	Yes
9. Were the exposure measures (independent variables) clearly defined, valid, reliable, and implemented consistently across all study participants?	Yes	Yes	Yes	Yes	Yes	Yes	Yes	Yes	Yes	Yes	Yes
10. Was the exposure(s) assessed more than once over time?	No	No	No	No	No	No	No	No	No	No	Yes
11. Were the outcome measures (dependent variables) clearly defined, valid, reliable, and implemented consistently across all study participants?	Yes	Yes	Yes	Yes	Yes	Yes	Yes	Yes	Yes	Yes	Yes
12. Were the outcome assessors blinded to the exposure status of participants?	NR	NR	Yes	NR	NR	Yes	NR	NR	NR	NR	NR
13. Was loss to follow-up after baseline 20% or less?	NA	NA	NA	NA	NA	NA	NA	NA	NA	NA	No
14. Were key potential confounding variables measured and adjusted statistically for their impact on the relationship between exposure(s) and outcome(s)?	Yes	No	No	No	No	Yes	Yes	Yes	Yes	No	No
Rating	Poor	Poor	Fair	Fair	Fair	Good	Fair	Poor	Fair	Fair	Good
